# One-stage knee replacement shows similar healing rates in patients with negative or positive preoperative cultures: a retrospective cohort study

**DOI:** 10.5194/jbji-10-237-2025

**Published:** 2025-07-25

**Authors:** Marta Sabater-Martos, Laura Morata, Josep Maria Segur, Alex Soriano, Juan Carlos Martínez-Pastor

**Affiliations:** 1 Orthopedic and Traumatology Department, Clínic Barcelona, Carrer Villarroel 170, 08036 Barcelona, Spain; 2 Department of Infectious Diseases, Clínic Barcelona, Carrer Villarroel 170, 08036 Barcelona, Spain; 3 IDIBAPS, CIBERINF CIBER in Infectious Diseases, University of Barcelona, Barcelona, Spain

## Abstract

**Introduction**: Treatment of chronic periprosthetic joint infections (PJIs) involves prosthesis removal, reimplantation, and antibiotic treatment. This process can be performed as a two-stage replacement or a one-stage replacement. One-stage replacement is classically performed only in patients who meet very strict criteria. The objective of this study was to analyse the healing and failure rates of one-stage knee replacement in patients with positive preoperative cultures and in those with negative preoperative cultures. Secondarily, we analysed the healing rate in patients with a sinus tract. **Material and methods**: We included 56 patients diagnosed with likely or confirmed PJI who underwent one-stage knee replacement in our centre between January 2016 and December 2021, with a minimum follow-up of 1 year. We evaluated the differences between cases with positive and negative preoperative cultures. Survival differences were assessed according to preoperative culture positivity and the presence of a sinus tract. **Results**: Preoperative cultures had positive results in 43 patients (76.8 %) and negative results in 13 patients (23.2 %). The overall failure rate was 12.5 % (seven patients), with one of these patients having had negative preoperative cultures. Of the 49 patients (87.5 %) with good results, 12 had negative preoperative cultures, and 37 had positive cultures ( p=1.00 ). Only 6 (10.7 %) of the 56 patients studied presented with a sinus tract. The differences in terms of healing and failure rates between patients with and without a sinus tract were not statistically significant ( p=0.57 ). **Discussion**: Using less strict criteria for patients, such as allowing preoperative negative cultures or the presence of a sinus tract, produced similar results to those for patients with only positive cultures or intact soft tissue.

## 1 Introduction

One of the most feared complications in prosthetic surgery is infection. For chronic periprosthetic joint infections (PJIs), treatment with curative intention consists of removal of the infected material with debridement followed by reimplantation and antibiotic treatment. One approach to this process is a two-stage replacement, in which prosthesis removal and reimplantation are performed separately after a variable period of antibiotic treatment. Another treatment strategy is the one-stage replacement, in which all steps are performed in a single surgical procedure, with subsequent antibiotic treatment. Until now, two-stage replacement has been considered to be the gold-standard treatment for PJI. However, in the last several years, there has been an increase in the indication for one-stage replacement, with several investigators arguing that a one-stage procedure is equally effective in treating PJI (Kunutsor et al., 2016; Razii et al., 2021; Nguyen et al., 2016; Pellegrini et al., 2021; Zahar et al., 2016). Further, the one-stage approach has the advantage of reducing costs, morbidity, and mortality compared with the two-stage replacement (Van Den Kieboom et al., 2021).

To date, one-stage replacement has been performed only in patients who met very strict criteria (Gehrke et al., 2016; Zahar et al., 2016; Zahar and Gehrke, 2016). In our centre, for knee PJIs, we have performed one-stage replacement in patients who do not meet some of these criteria, such as those showing the presence of negative cultures or a sinus tract that could be included in the surgical wound or resolved with a medial gastrocnemius flap.

We hypothesized that patients with negative cultures or sinus tracts do not have worse results after one-stage knee replacement compared with patients treated with a two-stage approach. The main objective of this study was to analyse the cure and failure rates of one-stage knee replacement in patients with negative cultures and in those with positive cultures. Our secondary objective was to analyse the cure rate of one-stage knee replacement in patients presenting with a sinus tract.

## 2 Material and methods

This retrospective study was approved by our ethical committee (approval no. CEIM:HCB/2023/0176). We obtained a list of all one-stage knee replacements performed in our centre between January 2016 and December 2021. We included all patients on whom a one-stage knee replacement was performed, with a minimum of 1 year of follow-up. For PJI classification, we used the European Bone and Joint Infection Society (EBJIS) classification (McNally et al., 2021). We excluded patients who had received partial replacement, two-stage replacement, or debridement with implant retention and aseptic replacement, as well as patients with a previous diagnosis of PJI. In our centre, one-stage knee replacement is performed in patients with negative cultures and in those with a sinus tract that could be included in the surgical wound or treated with a gastrocnemius flap. The gastrocnemius flap is considered for patients with a sinus tract that affects the distal part of the wound (below the patella) when direct closure is not possible. We do not perform one-stage replacement in patients with sepsis, important bone defects (i.e. patients needing femoral sleeves or cones for metaphyseal reconstruction), extensor mechanism deficiency, or important soft tissue problems that require microsurgery.

**Figure 1:**
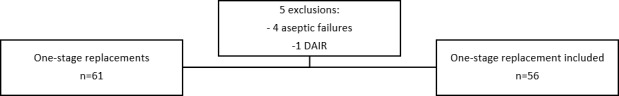
Sample selection. DAIR: debridement, antibiotics, and implant retention.

Using our informational codification system, we identified 61 patients on which a one-stage replacement had been performed. A total of 5 of these 61 were excluded due to incorrect codification: 4 were aseptic replacements, and 1 was a debridement with implant retention (Fig. 1). Our final sample was composed of 56 patients.

We obtained patients' epidemiological data, Charlson comorbidity index (CCI) scores, American Society of Anesthesiologists (ASA) grade, preoperative and intraoperative culture results, preoperative serological and synovial fluid results, and serological results during follow-up. We also recorded the appearance of any sinus tract and the need for a gastrocnemius flap during surgery. We considered treatment failure to be when additional surgeries for reinfection or recurrence were performed, when suppression treatment was necessary, or when death occurred due to the infection process (i.e. sepsis during surgery or follow-up). Patients were classified into two groups, depending on the preoperative culture results.

Our centre's protocol for a knee PJI is to perform a preoperative serological and synovial fluid analysis in the outpatient clinic. We send synovial fluid for biochemical and culture analysis in blood culture bottles (BCBs). If a patient is receiving antibiotic treatment, we stop it and postpone analysis for at least 2 weeks. If serological and/or synovial fluid results show suspicion of PJI, we perform a second knee arthrocentesis and analyse new cultures in BCBs using the polymerase chain reaction technique. If negative cultures persist, we classify the infection as preoperative culture negative. During surgery, we obtain synovial fluid by arthrocentesis prior to performing the arthrotomy. This fluid is sent for biochemical analysis in BCBs. After the arthrotomy, we also obtain four solid samples for conventional culture (two from synovial tissue and two from the implant–bone interface at the femur and tibia). We also collect two samples (tibia and femur) for histopathological analysis.

We performed a multivariate analysis to evaluate differences between preoperative culture-negative and culture-positive groups. The failure rate was represented by percentages. We compared survival curves on the basis of preoperative culture results and the presence of sinus tracts with a Chi-square test. Where necessary, a Fisher test was performed. We also calculated the agreement between the preoperative and postoperative EBJIS classification with the Cohen's kappa test. Statistical analysis was performed by using the Jamovi Project (2023) computer software (version 2.3, Sydney, Australia), and the statistical significance was set at p<0.05 .

## 3 Results

Of the 56 patients included, 21 were men (37.5 %), and 35 were women (62.5 %). A total of 23 surgeries were performed on left knees (41.1 %). The mean age was 73.8 years (SD of 8.7 years), with a mean body mass index of 30.9 kg cm^−2^. The mean comorbidity CCI was 4, and the mean ASA was II. The mean follow-up was 29.2 months (SD of 14 months). Prior to surgery, patients were classified into 19 likely infections (33.9 %) and 37 confirmed infections (66.1 %). Preoperative cultures were assessed in all patients: 43 had positive cultures (76.8 %), and 13 had negative cultures (23.2 %). Table 1 shows that the two groups were comparable.

**Table 1:** Epidemiological characteristics.

	Preoperative positive	Preoperative negative	p value
	cultures ( n=43 )	cultures ( n=13 )	
Age (years)	73.7 (SD 8.86)	73.92 (SD 8.52)	0.93
BMI (kg cm^−2^)	30.85 (SD 4.58)	31.7 (SD 6.76)	0.89
ASA	2.14	2.36	0.17
CCI	3.70	4.07	0.34
Preoperative EBJIS group			
– Likely infection	12 (27.9 %)	7 (53.8 %)	0.083
– Confirmed infection	31 (72.1 %)	6 (46.2 %)	

BMI: body mass index.

**Figure 2:**
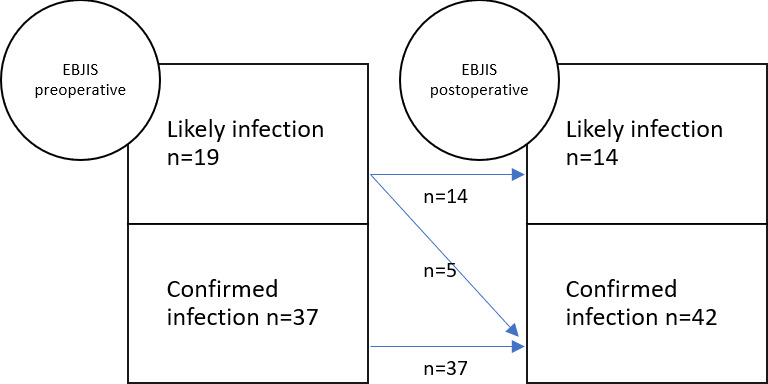
PJI classification before and after surgery.

After surgery, five patients from the likely infection group were transferred to the confirmed infection group due to the presence of two positive cultures for the same microorganism. All five patients had presented with positive preoperative cultures. Of the 13 patients with negative preoperative cultures, 7 belonged to the likely infection group preoperatively and did not change groups after surgery. Their intraoperative cultures remained negative. The other six patients with negative cultures had been classified as having confirmed infections preoperatively. Only one of the six tested positive for *Staphylococcus epidermidis* in more than two samples. The other five patients had negative intraoperative culture results. Postoperatively, patients were reclassified as follows: 14 patients were in the likely infection group (25 %), and 42 were in the confirmed infection group (75 %) (Fig. 2). Cohen's kappa coefficient was 0.79 (95 % confidence interval (CI) of 0.61–0.96).

The failure rate was 12.5 % (seven patients): one patient had negative preoperative and intraoperative cultures, and six had positive preoperative cultures. The success rate was 87.5 % (49 of 56). In preoperative culture-negative cases, the success rate was 92 % (95 % CI of 78.9 %–100 %) (12 of 13) vs. 86 % (95 % CI of 76.3 %–97.1 %) (37 of 43) when preoperative cultures were positive (hazard ratio of 1.87 (95 % CI of 0.22–15.5, p=1.00 )). Two patients failed due to the need for suppressive therapy, and five failed due to reinfection and the need for a two-stage revision.

In total, 6 (10.7 %) of the 56 patients had initially presented with sinus tracts. All patients with sinus tracts had positive cultures (five *S. epidermidis* and one *S. aureus*). Only 1 of the 6 patients with sinus tracts failed (17 %) vs. 6 of the 50 (12 %) in the non-sinus-tract group ( p=0.57 ). Three patients received gastrocnemius flaps; none of these three failed (Table 2). The patient with a sinus tract and failure (need for suppressive antibiotic therapy) had had positive preoperative cultures for *S. epidermidis* and did not receive a gastrocnemius flap.

**Table 2:** Differences in terms of healing and failure depending on culture results and sinus tract presence.

	Healing ( n=49 , 87.5 %)	Failure ( n=7 , 12.5 %)	p value
Cultures			
– Positive ( n=43 )	37	6	1.00
– Negative ( n=13 )	12	1	
Sinus ( n=6 , 10.5 %)	5	1	0.57
EBJIS			
– Likely	13	1	0.66
– Confirmed	36	6	

## 4 Discussion

The most important finding of this study was that the use of less strict criteria for patients, such as allowing the presence of negative cultures or a sinus tract, showed similar results to those in patients with negative cultures or intact soft tissue in one-stage knee replacement. This finding suggests that the classic criteria, whereby the infecting microorganism needs to be determined (Gehrke et al., 2013), can be expanded to patients with negative cultures and those who present with a sinus tract that can be included in the surgical wound or resolved by means of a gastrocnemius flap.

The gold-standard treatment for chronic PJI has been two-stage replacement, with cure rates of 80 %–90 % and reinfection rates of 15 % (Bongers et al., 2020; Mahmud et al., 2012). This pathological condition and treatment entail high economic costs, as well as high morbidity and mortality (Van Den Kieboom et al., 2021). For all these reasons, one-stage replacement has emerged as an increasingly accepted strategy for the treatment of chronic PJI. Historically, this type of surgery was solely performed on strictly selected patients (Gehrke et al., 2013, 2016). However, several studies have already shown no significant difference in results between patients undergoing one-stage replacement and those undergoing two-stage replacement (Van Den Kieboom et al., 2021; Kunutsor et al., 2016; Pellegrini et al., 2021; Tuecking et al., 2021; Zahar et al., 2016). Other studies that encompassed a broader selection of patients and included those with negative cultures in one-stage replacement have reported similar results. Razii et al. (2021), for example, analysed 84 patients who had undergone one-stage replacement without excluding those with negative cultures and obtained a cure rate of 90.5 %.

Castellani et al. (2017) studied the factors that may influence the decision to use one-stage replacement vs. two-stage replacement. They included 110 patients, of whom 35 (32 %) underwent one-stage replacement and 75 (68 %) underwent two-stage replacement. They observed no difference between groups in terms of sex, age, or time since previous intervention or microorganism; surgeons were more likely to perform one-stage replacement in patients who were also undergoing hip surgery, those with a history of chronic renal failure, and those with negative cultures. This observation made us wonder whether the classic criteria really conform with reality. In the 56 cases of one-stage replacement examined in our study, we found that 13 had had negative cultures and that the results did not differ significantly from those of patients with positive cultures: the cure rates were 86 % for patients with positive cultures vs. 92 % for patients with negative cultures.

In contrast with the results of other published studies, in our study, we obtained a preoperative and postoperative agreement coefficient (using the EBJIS classification) of 0.79, with a 25 % rate of postoperative likely infection patients. In a 2003 study, Sousa et al. (2023) analysed 361 prosthesis revisions and concluded that the EBJIS classification showed an agreement between preoperative and postoperative patients of 0.9 (95 % CI of 0.8–0.9). These differences between study results could be explained by our small sample size and the exclusion of aseptic replacement cases.

Two limitations of our study should be acknowledged. First, this is a retrospective study, which comes with its own inherent limitations. Second, our small sample size could explain the differences between our findings and those in the literature.

It appears to be the case that using less strict inclusion criteria for one-stage knee replacement, such as allowing cases with culture-negative infections or sinus tracts, yields results that are comparable to those observed in patients undergoing the same procedure with positive cultures or intact soft tissue.

## Data Availability

Software code and data supporting the conclusions of this article can be made available by the authors upon request.

## References

[bib1.bib1] Bongers J, Jacobs AME, Smulders K, van Gijs G, Goosen JHM (2020). Reinfection and re-revision rates of 113 two-stage revisions in infected TKA. J Bone Joint Infect.

[bib1.bib2] Castellani L, Daneman N, Mubareka S, Jenkinson R (2017). Factors Associated with Choice and Success of One- Versus Two-Stage Revision Arthroplasty for Infected Hip and Knee Prostheses. HSS Journal.

[bib1.bib3] Gehrke T, Zahar A, Kendoff D, Gehrke T, Zahar A, Kendoff AD (2013). One-stage exchange IT ALL BEGAN HERE. Bone Joint J.

[bib1.bib4] Gehrke T, Alijanipour P, Parvizi J, Gehrke T, Alijanipour P, Fellow R, Parvizi J (2016). The management of an infected total knee arthroplasty. Clin Orthop Relat R.

[bib1.bib5] Kunutsor SK, Whitehouse MR, Lenguerrand E, Blom AW, Beswick AD, Strange S, Garfield K, Gooberman-Hill R, Moore D, Burston A, Simon J, King G, Wylde V, Noble S, Lane A, Carroll F, Webb J, MacGowan A, Jones S, Taylor A, Dieppe P, Toms A, Wilson M, Stockley I, Burston B, Whittaker JP, Board T (2016). Re-infection outcomes following one- and two-stage surgical revision of infected knee prosthesis: A systematic review and meta-analysis. PLOS One.

[bib1.bib6] Mahmud T, Lyons MC, Naudie DD, MacDonald SJ, McCalden RW (2012). Assessing the Gold Standard: A Review of 253 Two-Stage Revisions for Infected TKA. Clin Orthop Relat R.

[bib1.bib7] McNally M, Sousa R, Wouthuyzen-Bakker M, Chen AF, Soriano A, Vogely HC, Clauss M, Higuera CA, Trebše R (2021). The EBJIS definition of periprosthetic joint infection. Bone Joint J.

[bib1.bib8] Nguyen M, Sukeik M, Zahar A, Nizam I, Haddad FS (2016). One-stage Exchange Arthroplasty for Periprosthetic Hip and Knee Joint Infections. Open Orthop J.

[bib1.bib9] Pellegrini A, Meani E, Macchi V, Legnani C (2021). One-stage revision surgery provides infection eradication and satisfying outcomes for infected knee arthroplasty in selected patients. Expert Rev Anti-Infe.

[bib1.bib10] Razii N, Clutton JM, Kakar R, Morgan-Jones R (2021). Single-stage revision for the infected total knee arthroplasty. Bone Jt Open.

[bib1.bib11] Sousa R, Ribau A, Alfaro P, Burch M-A, Ploegmakers J, McNally M, Clauss M, Wouthuyzen-Bakker M, Soriano A (2023). The European Bone and Joint Infection Society definition of periprosthetic joint infection is meaningful in clinical practice: a multicentric validation study with comparison with previous definitions. Acta Orthop.

[bib1.bib12] Tuecking L-R, Silligmann J, Savov P, Omar M, Windhagen H, Ettinger M (2021). Detailed Revision Risk Analysis after Single- vs. Two-Stage Revision Total Knee Arthroplasty in Periprosthetic Joint Infection: A Retrospective Tertiary Center Analysis. Antibiotics.

[bib1.bib13] Van Den Kieboom J, Tirumala V, Box H, Oganesyan R, Klemt C, Kwon Y-M (2021). One-stage revision is as effective as two-stage revision for chronic culture-negative periprosthetic joint infection after total hip and knee arthroplasty. Bone Joint J.

[bib1.bib14] Zahar A, Gehrke TA (2016). One-Stage Revision for Infected Total Hip. Arthroplasty.

[bib1.bib15] Zahar A, Kendoff DO, Klatte TO, Gehrke TA (2016). Can Good Infection Control Be Obtained in One-stage Exchange of the Infected TKA to a Rotating Hinge Design? 10-year Results. Clin Orthop Relat R.

